# Controlling the cortical actin motor

**DOI:** 10.1007/s00709-012-0403-9

**Published:** 2012-04-15

**Authors:** Julie Grantham, Ingrid Lassing, Roger Karlsson

**Affiliations:** 1 Department of Chemistry and Molecular Biology, University of Gothenburg, Gothenburg, Sweden; 2 Department of Cell Biology, Wenner-Gren Institute, Stockholm University, SE-106 91 Stockholm, Sweden

**Keywords:** Cell motility, Actin dynamics, Actin-binding proteins, Actin folding, Molecular chaperone, CCT

## Abstract

Actin is the essential force-generating component of the microfilament system, which powers numerous motile processes in eukaryotic cells and undergoes dynamic remodeling in response to different internal and external signaling. The ability of actin to polymerize into asymmetric filaments is the inherent property behind the site-directed force-generating capacity that operates during various intracellular movements and in surface protrusions. Not surprisingly, a broad variety of signaling pathways and components are involved in controlling and coordinating the activities of the actin microfilament system in a myriad of different interactions. The characterization of these processes has stimulated cell biologists for decades and has, as a consequence, resulted in a huge body of data. The purpose here is to present a cellular perspective on recent advances in our understanding of the microfilament system with respect to actin polymerization, filament structure and specific folding requirements.

## Introduction

### The treadmilling actin motor—an essential force generator

Motility is a fundamental property of virtually all cellular events including transport of intracellular components, cell migration during development and tissue maintenance. It also has a major impact upon the behavior of cells during different pathological conditions including cancer.

The microfilament system at the cell periphery intimately associates with the plasma membrane, making this interface between the cell interior and its outside world an exquisitely dynamic sensory organelle developed for information transfer. In so-called lamellipodia and filopodia, at the advancing edge of migrating cells, actin builds dense arrangements (Hoglund et al. 1980; Koestler et al. 2008; Small 1981) of dynamic filaments and accessory proteins that undergo complex patterns of remodeling in response to signals relayed by surface receptors (Lindberg et al. 2008; Pollard and Borisy 2003). Thus bundles and sheet-like arrangements of actin filaments form protrusions that explore and respond to the external milieu during normal as well as pathological conditions requiring cell migration.

**Fig. 1:**
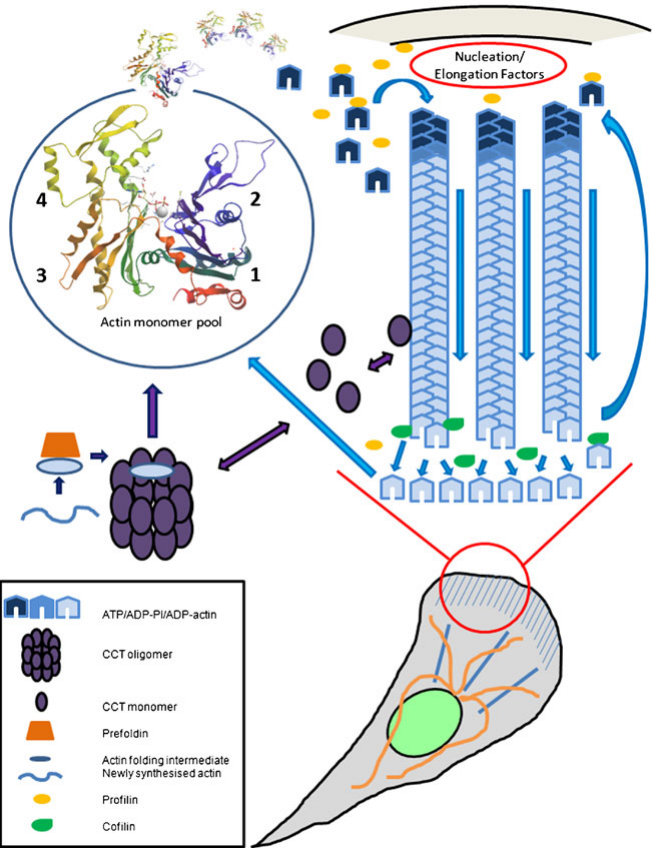
The dynamic actin motor. The flow of actin molecules through the filamentous and monomeric pools and the input of newly folded actin into the system from the prefoldin/CCT-apparatus are illustrated. Blue and purple arrows indicate actin treadmilling and folding pathways/chaperone interactions, respectively. The actin within the circle represents the total pool of nonfilamentous actin and numbers denote subdomain organization. For simplification numerous interactions with binding proteins dealt with in the text are omitted. Note that CCT is contributing to the actin system both as a folding machine and via subunit interactions with filamentous actin. The actin structure is that determined for the open form of profilin:actin (Chik et al. 1996; pdb: 1HLU). Molecules and the plasma membrane are not drawn to scale

The actin molecule is a bi-lobed structure with four subdomains (SD 1–4) surrounding a central Mg^2+^-nucleotide binding cleft (Kabsch et al. 1990) and Fig. 1. This overall fold defines a structural superfamily of proteins which include several sugar kinases, heat shock proteins, actin related proteins (Arps) in eukaryotes and the more distant actin-like proteins in prokaryotes (Bork et al. 1992; Dominguez and Holmes 2011; Flaherty et al. 1991; Rivera et al. 2011; Robinson et al. 2001; van den Ent et al. 2001). The actin subdomain organization is formed by the discontiguous arrangement of the polypeptide chain that starts in SD1 and then passes through SD2, SD1 SD3, SD4, SD3 and finally back into SD1. Consequently the structures of the individual SDs of the actin molecule are highly interdependent, forming a molecular organization distinct from many multi-domain proteins which consist of consecutively arranged modules along the protein chain in a beads-on-a-string format. The divalent cation-nucleotide complex (Ca^2+^/Mg^2+^-ATP/ADP) at the central cleft is essential for the structural stability of the molecule as a whole and hence is directly integrated with the functional status of the protein.

Notably, the microfilament system is under constant flux; the overall filament arrangement is continuously remodeled and within individual filaments there is a rapid turnover of subunits. Actin molecules carrying ATP are incorporated at membrane-facing fast polymerizing (+)-ends of the filament and then successively flow through the structure towards the inward pointing (−)-end where dissociation of ADP-actin occurs. This translocation of subunits through the filament is commonly referred to as treadmilling (Wegner 1976), and occurs both in vitro and in vivo (Bugyi and Carlier 2010; Lai et al. 2008; Okabe and Hirokawa 1989; Wang 1985). As a consequence of treadmilling, the filament has a biochemical asymmetry defined by the nucleotide content of its subunits in addition to its structurally determined polarity. The actin system with regard to folding, the monomer pool, polymerization and treadmilling is summarized in Fig. 1.

The actin-bound ATP is hydrolysed following incorporation at the (+)-end, and the phosphate group (Pi) subsequently dissociates. The ADP-actin molecules released from the (−)-end need to be recharged with ATP before they can participate in a new cycle of filament formation (Korn et al. 1987; Schűler 2001). The high numbers of treadmilling filaments in lamellipodia and filopodia therefore account for a considerable portion of the actin-dependent ATP-consumption, which by itself represents a major portion, possibly as much as 50 % of total ATP consumption in non-muscle cells during homeostasis (Bernstein and Bamburg 2003).

The concentration of monomeric actin (G-actin) that coexists with filamentous actin (F-actin) under steady state treadmilling is referred to as the critical concentration (Cc) and is the consequence of the K_d_:s characterizing monomer affinity for the two filament ends. Under physiological salt conditions in vitro this concentration is around 0.1 μM (e.g. Korn 1982). However, in the cell, the situation is far more complex due to the presence of many actin-binding proteins that influence growth, structure and organization of the filaments and thereby allow for spatiotemporal control of the system and its force-generation. In the lamellipod, the concentration of F-actin is estimated to range from 500 μM to 1600 μM and that of monomeric actin to be at least 150 μM (see Koestler et al. 2009), and it is here where many actin regulatory components operate to fine-tune actin remodeling and thereby impact upon motility of the leading edge.

Classically the WASP, WAVE, Ena/VASP and the formin family of proteins have been identified as major responders to signals that are relayed from the cell surface by various receptor molecules to the microfilament system (Campellone and Welch 2010; Dominguez 2010; Goley and Welch 2006; Renault et al. 2008; Takenawa and Suetsugu 2007). Their concerted action enables the site-directed nucleation and elongation of actin filaments required in response to the signalling. These molecules, commonly referred to as actin nucleation and elongation promoting factors (NEPFs), in turn cooperate with members of the β-thymosin (BT), profilin, and actin depolymerizing factor/cofilin-homology (ADFH)-families, to name a few, to modulate actin activities such as nucleotide binding and hydrolysis, nucleation (i.e. initiation of filament formation) and elongation as well as steady-state filament subunit turnover (e.g. Dominguez and Holmes 2011). Thus the receptor signalling is transduced into motion by the activity of actin under the control of a multitude of accessory components. Due to this complex array of protein interactions and structural reorganizations, the knowledge gathered from experiments with isolated components in vitro may not always be fully relevant when considering the finely tuned control of actin filament turnover in vivo.

Despite cellular conditions favoring polymerization, a varying but substantial fraction of actin remains unpolymerized (Blikstad and Carlsson 1982) due to its interaction with monomer binding proteins. A major portion of this is polymerization-blocked due to interactions with β-thymosin (Carlier et al. 1993; Safer et al. 1991). Another monomer actin pool consists of profilin:actin. Whilst this is a nucleation-inhibited actin the complex still can dock with existing filament (+)-ends promoting filament elongation after dissociation of the profilin (Korenbaum et al. 1998; Nyman et al. 2002; Pantaloni and Carlier 1993; Tilney et al. 1983). Consequently, the profilin:actin complex represents a dynamic organization of non-filamentous actin rather than a stably sequestered reservoir (Grenklo et al. 2003; Hajkova et al. 2000). The fact that profilin-bound actin readily replaces ADP for ATP (Korenbaum et al. 1998; Mockrin and Korn 1980) further emphasizes this role of profilin:actin as an actin captured “in transit” to filament assembly either after dissociation from filament (−)-ends or after release from more stable storage forms. The two structures of profilin:actin with the actin in “open” and “tight” conformations, respectively, (Chik et al. 1996; Schutt et al. 1993) have now been complemented with a detailed analysis of the influence of profilin upon the actin structure, which demonstrated actin domain rotations and a key-role for residue W356 during profilin-induced actin nucleotide exchange (Porta and Borgstahl 2012). A profilin-like function with respect to the ability of nucleation-inhibited docking of new actin subunits to filament (+)-ends has also been shown for Wiscott-Adrich Syndrome Homology 2 (WH2)-domains (Dominguez 2007; Mattila et al. 2003; Yarar et al. 2002) as well as for some other non-profilin family proteins (Boquet et al. 2000; Hertzog et al. 2004; Hertzog et al. 2002; Husson et al. 2010).

Insights from structural and biochemical studies have revealed extensive similarities between the canonical WH2 domain in WASP, the GAB domain in Ena/VASP and the peptide members of the β-thymosin family (Carlier et al. 2011; Dominguez 2007). This has led to the identification of the WH2 domain, more recently referred to as the BT/W-domain, (e.g. Didry et al. 2011), as a signature for the control of actin polymerization along with the profilin family of monomer actin binding molecules. The BT/W-domain is found in a large number of proteins, sometimes in association with other domains such as the Arp2/3 binding domain (CA for connector-acidic) in WASP and sometimes in a repeated arrangement such as in Spire and Cobl (Carlier et al. 2011). Subtle variations in this actin binding module, that adjust the interaction with actin molecules have dramatic effects on actin control, resulting in either stably sequestered actin as in the case of β-thymosin, or in a complex that can add the actin to the (+)-end of the filament in a profilin-like action. In a recent study employing site-directed mutagenesis, biochemical and structural analyses Didry et al. (2011) characterized the molecular details behind this versatile behavior of the BT/W-domain and demonstrated the importance of its N-terminus and so-called linker sequence for its effect on actin.

The BT/W-domains often operate in multi-modular proteins together with the Arp2/3 complex to initiate actin nucleation downstream a variety of signaling cues, involving RhoGTPases and PIPs (Campellone and Welch 2010; Takenawa and Suetsugu 2007), and sometimes, as in the case of N-WASP may directly cooperate with VASP (Castellano et al. 2001; Yarar et al. 2002) or associate with microtubules (Rottner et al. 2010). Currently it is unclear if profilin:actin constitutes the major source of polymerization-competent actin for filament elongation in this context. Following reduction in profilin levels by siRNA, cells still have capacity for edge advancement and to migrate, though with an altered activity that appears to be cell line-dependent (Ding et al. 2006; Janke et al. 2000; Zou et al. 2007). However, introducing non-dissociable profilin:actin into cells by microinjection dramatically blocks actin-dependent motility demonstrating that profilin:actin plays an essential role in coordinating actin dynamics (Grenklo et al. 2003; Hajkova et al. 2000).

In the case of the archetypical formin family members (mDia in mammalian cells and cdc12 in *S.pombe*), profilin:actin is the source for site-specific filament elongation (Chang et al. 1997; Paul and Pollard 2009; Watanabe et al. 1997). The recruitment of profilin:actin by these proteins depends on profilin binding to the polyproline stretch located in the formin homology1 domain (Kursula et al. 2008), the availability of which for profilin:actin binding is under control of Rho. Notably also N-WASP, WAVE and Ena/VASP have the capacity to bind profilin/profilin:actin via similar proline-mediated interactions (Kursula et al. 2008; Miki et al. 1998; Reinhard et al. 1995; Suetsugu et al. 1998), but are not strictly dependent on profilin for optimal elongation when studied in vitro (Breitsprecher et al. 2011; Hansen and Mullins 2010). In vivo where actin molecules are under repeated cycles of treadmilling, the enhancement of actin nucleotide exchange by profilin ensures that the actin incorporated into filaments is an ATP-actin. Although the ATP-concentration by far exceeds that of ADP in vivo, local variations in ATP-concentration occurring transiently could include ATP-depletions under which profilin-spurred enhancement of nucleotide exchange on the actin may be important. However, we rather favor the view that this function of profilin operates as a “check-point”, ensuring that the actin molecule transferred to the filament (+)-end for elongation indeed carries ATP and not ADP, which is a prerequisite for maintenance of the treadmilling kinetics.

Furthermore, actin filament binding proteins recognize distinct subunit structures directly related to the status of their bound nucleotide. For instance, when activated by association with a NEPF such as N-WASP or WAVE, the heptameric Arp2/3 complex containing 2 actin related molecules will bind to ATP/ADP.Pi-carrying subunits of a preexisting (mother) filament where it initiates formation of a new (daughter) filament “branch” at a 70° angle (Mullins et al. 1998; Robinson et al. 2001). Observation of such branches in vitro (Mullins et al. 1998) as well as in vivo (Svitkina and Borisy 1999) led to the so-called dendritic branch model for polymerization driven lamellipodial advancement (Pollard et al. 2000). Separate studies using electron microscopy and different protocols for specimen preparation to visualize the lamellipodial actin organization either demonstrated the presence (Svitkina and Borisy 1999) or absence (Hoglund et al. 1980; Small 1981) of such branches, and have spurred intensive work to understand to what extent these structures existed in vivo (e.g. Small 2010; Urban et al. 2010). This issue has now been settled by electron tomography and image analysis (Ydenberg et al. 2011), demonstrating that branched actin filament arrangements are present in the lamellipodia of migrating cells, albeit to a much lesser extent than what was commonly anticipated from the original model (Small et al. 2011; Yang and Svitkina 2011). Consequently, the question is if in vivo the majority of the Arp2/3 branches actually de-branch in direct succession to nucleation, leaving the Arp2/3-complex to move inwards with the pointed end of the filament (Lai et al. 2008). Interestingly, the branches observed are distributed in a relatively scattered pattern across the lamellipod possibly representing remnants of such structures remaining after treadmilling. Therefore it seems plausible that repeated branching/de-branching is frequently occurring at the tip of the advancing lamellipod where it contributes to edge advancement. Final proof for this will have to await further development of specimen preparation and electron microscopy techniques that can resolve protein organization directly at the inner leaflet of the plasma membrane. Notably, the Arp2/3-complex is dispensable for fibroblast motility in culture although cell morphology, behavior and responsiveness to signaling cues are strongly affected in its absence (Wu et al. 2012).

In the lamellipod of advancing cells, actin subunits that dissociate during treadmilling from the inward pointing (−)-ends are rapidly recycled back to the front to contribute to the elongation. Consequently two tightly coupled but counter-directed “streams” of actin, one formed by treadmilling the filament subunits inwards and the other by recycling actin monomers to the front coexist in this highly confined space at the cell periphery. Whilst ATP-hydrolysis upon subunit incorporation powers the inward flow of actin molecules, much less is known concerning how the outward-directed flow occurs to maintain the extreme actin concentrations required at the tip of lamellipodia and filopodia for their steady-state advancement. Consider for instance a segment of a lamellipod consisting of say 100 filaments or a filopod with a core of 20 filaments. For these structures to protrude over the substratum at a speed of 5 μm/min, in total 3100 and 775 actin molecules, respectively, would need to be added per second to these filament arrangements to support the elongation. It is generally believed that free diffusion cannot make up for these numbers; instead the contribution by the activity of different myosins appears more likely (Kerber et al. 2009; Keren et al. 2009; Koestler et al. 2009; Zicha et al. 2003). It is also interesting to note that this flow of monomeric actin towards the tip of these membrane protrusions, at least in the case of the filopodia, must be excluded from the core of densely packed treadmilling filaments building these structures and therefore occurs in the juxtamembrane space. Furthermore, the movement of unconventional myosins with cargos like integrins, VASP and cadherins (Arjonen et al. 2011) makes it plausible that active transport also plays a role in forward directed flow of polymerization-competent actin.

The ADF/cofilins are the archetype members of the ADFH family of proteins and they are tightly associated with the control of actin at filament (−)-ends at the inner part of the lamellipod. Initially, this group of proteins was identified as preferentially binding ADP-actin monomers and accelerating filament depolymerization by capturing actin subunits dissociating form the filament (−)-end. They were later found to also bind directly to and sever filamentous actin (Bamburg 1999; Poukkula et al. 2011). The subsequent transfer of the ADF/cofilin-bound ADP-actin to profilin and formation of polymerization competent ATP-actin as in profilin:actin has generally been considered to be an effect of mass-action due to the variation in K_d_-values, shifting the actin towards the latter complex. However, recent observations suggest that this process may be more complex and involve the activity of other proteins such as cyclase associated protein (CAP/Srv2) and coronin which both interact with ADF/cofilin and impact significantly on actin dynamics (Chan et al. 2011; Chaudhry et al. 2010; Moriyama and Yahara 2002). The ADFH family, in addition to ADF/cofilin, is known to consist of 4 subclasses of structurally related protein modules (Poukkula et al. 2011) all of which preferentially recognize ADP-bound actin filament subunits and therefore bind “older” parts of the filament. Interestingly one member of this family, instead of binding filamentous actin, targets Arp2/3-branches and causes their dissociation from the mother filament (Gandhi et al. 2010).

**Fig. 2:**
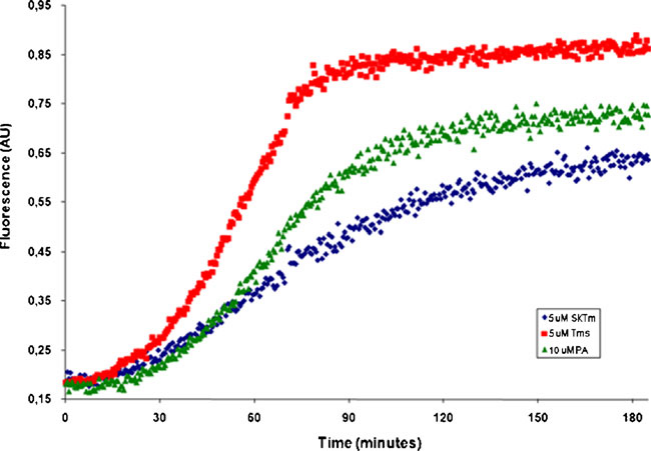
Tropomyosin isoforms have distinct effects upon actin polymerizing from profilin:actin. Calf profilin:actin (10 μM) was incubated with tropomyosin isoforms (5 μM) and pyrenyl labeled β-actin (10 nM) under polymerizing salt conditions and the increase in pyrenyl fluorescence was monitored. Typically, the polymerization rate is faster (roughly 3 times) and the final steady-state level of polymerization higher in the presence of the non-muscle isoform Tm5 compared to skeletal muscle tropomyosin. Calf thymus profilin:actin was isolated as described (Schűler et al. 2006) and the tropomyosin isoforms were prepared as in (Hillberg et al. 2006; Lassing et al. 2010). Red squares, non-muscle Tm5; green triangles, profilin:actin only; blue diamonds, skeletal muscle Tm

In addition to components that support the treadmilling and recycling of the actin, there is an array of proteins organizing filaments into the different arrangements typical of lamellipodia and filopodia. Often these are important for providing connections with the plasma membrane. Fimbrin, α-actinin, filamin and dystrophin are such examples, representing a superfamily of modular proteins with an array of interaction partners involved in transferring chemomechanical information between the actin microfilament and surrounding tissue. They express actin filament bundling and/or cross-linking activities and are typically controlled by Ca^2+^-ions, phospholipid binding and phosphorylation (Le Clainche and Carlier 2008; Matsudaira 1994; Nakamura et al. 2011; Otey and Carpen 2004; Sjoblom et al. 2008). Certain non-muscle isoforms of tropomyosin seem to play a special role in this context. It is well known that in skeletal muscle, tropomyosin associates alongside the thin filaments and cooperates with the troponin-complex to control actomyosin interaction in response to Ca^2+^-ions. However, in non-muscle cells, the organization of tropomyosin is much more complex, with a large number of isoforms that appear to distribute in distinct patterns between the internal, less dynamic filament arrangements, and the constantly remodeled actin organization at the cell periphery. Interestingly, there appears to be a connection between tropomyosin isoform expression, malignancy and motility (e.g. O’Neill et al. 2008). Furthermore, non-muscle tropomyosin in addition to being associated with actin filaments seems to assemble into non-filament bound “particles” that may represent storage forms from which the protein can be recruited when needed (Grenklo et al. 2008; Lassing et al. 2010; Lindberg et al. 2008). Contrary to the long existing view that non-muscle tropomyosins predominantly distribute in areas of the cell distant to the edge, it now seems that the presence of tropomyosin isoforms extends all the way to the tip of lamellipodia (Hillberg et al. 2006). This agrees with observations pointing to a role for tropomyosin during formin-controlled actin polymerization (Evangelista et al. 2002; Skau et al. 2009; Tojkander et al. 2011; Wawro et al. 2007). It is therefore interesting that skeletal muscle tropomyosin interferes with actin polymerization in vitro when initiated from profilin:actin while the non-muscle isoform TM5, in contrast, increases filament formation under the same conditions, Fig. 2. Though a molecular explanation to this result remains to be established, it appears to reflect distinct accessibilities for profilin:actin to dock and deliver new actin subunits at filament (+)-ends, depending on the filament-bound tropomyosin isoform. This in turn could be due to different interaction properties of the two TM-isoforms with filamentous actin, causing a variation in subunit structure that propagates into a fine-tuning of the docking-surface for profilin:actin. Together with the fact that tropomyosin can compete with ADF/cofilin and N-WASP for filament binding (Blanchoin et al. 2001; Ono and Ono 2002), this indicates that the classical view of tropomyosins as primarily filament stabilizing components does not completely describe the function of this family of actin binding proteins in non-muscle cells (see also Bugyi et al. 2010; Grenklo et al. 2008; Lassing et al. 2010). This is a rapidly expanding field with potential implications for drug development and chemotherapy to target tropomyosin action in malignant cells (O’Neill et al. 2008; Schevzov et al. 2011; Stehn et al. 2006).

Many of the NEPFs, and several other components controlling actin turnover at the periphery such as WASP, WAVE, profilin and cofilin directly bind the anionic phosphatidylinositol lipids [PI(4,5)P_2_, PI(3,4)P_2_ and PI(3,4,5)P_3_]. These interactions provide an explanation for the accumulation of many actin organizing proteins at the inner leaflet of the membrane and play important regulatory mechanisms influencing both actin turnover and the metabolism of the phosphatidylinositol lipids (e.g. Arjonen et al. 2011; Hilpela et al. 2004; Karlsson and Lindberg 2007; van Rheenen et al. 2007; Zhang et al. 2012). The spectrin, ankyrin and ezrin-radexin-moesin families of proteins represent other components long recognized to be of major importance in this context (Bennett and Healy 2008; Neisch and Fehon 2011). This is also the case for the integrin family of transmembrane receptors. These latter proteins are pivotal for cell-extra cellular matrix (ECM) attachments where at the cytoplasmic side of the plasma membrane they form the core of the adhesome (Geiger and Yamada 2011). At the tip of lamellipodia and filopodia the integrins screen the ECM for new interactions while surfing on elongating actin filaments (Galbraith et al. 2007). They reach their position at the very edge by the activity of an unconventional myosin (Zhang et al. 2004) that by itself is recruited and activated at the membrane by PI(3,4,5)P_3_ after transport and release from rab-containing vesicles along microtubules (Arjonen et al. 2011). The similar process of an unconventional myosin-dependent transportation to the front of the advancing cell edge seems to be responsible also for the localization of Ena/VASP and certain cadherins (Almagro et al. 2010; Tokuo and Ikebe 2004) as mentioned above.

The more recently discovered MIG-10/RIAM/lamellipodin (MRL) protein family is also operating at the inner side of the plasma membrane. These proteins specifically bind the phosphatidyl inositol lipid PI(3,4)P_2_ through a PH-domain and Ena/VASP through a central FPPPP-sequence motif (Krause et al. 2004; Lafuente et al. 2004; Michael et al. 2010). Furthermore, MRL-proteins are controlled by RasGTP (Depetris et al. 2009; Jenzora et al. 2006) and therefore represent another node between growth factor signaling and the control of the microfilament system, and lamellipodin has been shown to operate in dorsal ruffles and during axonal morphogenesis (Michael et al. 2010). Its activation at the membrane and subsequent recruitment of Ena/VASP appears to be negatively controlled by profilin (Bae et al. 2010), which may be one reason why profilin can act as a tumor suppressor by reducing migration of certain cancer cells (Janke et al. 2000; Zou et al. 2007) despite its role to provide new actin molecules to growing filament (+)-ends through NEPF-recruitment of profilin:actin as discussed above. The other major member of this family, RIAM, on the other hand appears to operate in the adhesome to activate tallin and subsequently integrin function downstream of RapGTP (Lafuente et al. 2004; Lee et al. 2009), as well as playing an important role in T-cell receptor signalling (Patsoukis et al. 2009). Also this protein contains polyproline sequence motifs that can potentially mediate interaction with profilin in addition to Ena/VASP, and recently RIAM was linked to invasion and growth control of a human melanoma cell line (Hernandez-Varas et al. 2011). Thus the MRL-family of proteins contribute to the intricate pattern of signaling pathways that operates downstream of surface receptors to control cell motility by directly influencing components intrinsic to actin motor control.

### The actin filament—a plastic structure intrinsic to the motor control

Despite the fact that the structure of the actin filament at high resolution so far has not been possible to determine (Splettstoesser et al. 2011) it is well established from several studies e.g. (Galkin et al. 2012, 2010; Michelot and Drubin 2011; Oda and Maeda 2010) that the filament subunits are subject to conformational changes due to nucleotide hydrolysis in conjunction with their incorporation into the growing filament. As touched upon in conjunction to the ADFH and TM families above, interactions with different filament binding proteins also influence the filament structure. This has for instance been reported for myosin and gelsolin (Prochniewicz et al. 1996; Uyeda et al. 2011) which upon binding cause subunit structure modulations that seem to propagate for various distances along the filament, and, at least in the case of myosin, results in an increased binding affinity during subsequent interactions. In view of the constrained structure due to the intra-subunit interactions forming the filament, this plasticity is both intriguing and astonishing, and again emphasizes the importance that the incoming actin at the (+)-end is loaded with ATP. Not only is this a prerequisite for maintaining the directional turnover of actin molecules, i.e the treadmilling phenomenon, but also for the fine-tuning of these interactions and hence the distribution of different filament binding components at the cell periphery. Furthermore, in several cases such as the Arp2/3-complex and the ADF/cofilin family members, filament interactions depend on the subunit-bound nucleotide (see also Ito et al. 2011).

Recent progress in cryo-electron microscopy has enabled reconstructions of the filament structure at a resolution approaching 6.5 Å and has allowed for *in silico* fitting of the actin monomer structure into the polymer (Fujii et al. 2010). This approach suggests that extensive transitions of subdomain orientation are necessary to accommodate the actin molecule (i.e. ADP-actin) within the polymer envelope. No doubt, future improvements of this technique will enable an even better understanding of the relationship between polymerization-competent monomers and filamentous actin with respect to structure. However, the high degree of plasticity combined with the influence of filament binding proteins on actin subunit structure suggest that extensive work remains before we can reach a full understanding of the different filament conformations that are likely to occur in the cell. It may turn out that actin filaments captured in vitro, in the absence of other protein components that in vivo influence polymerization and filament turnover and function, in fact have a structure that does not fully represent the cellular situation.

The delicate structural transitions occurring in the “living” actin filament together with treadmilling and interactions with numerous actin-binding proteins have placed strong evolutionary constraints on the actin molecule explaining why so few naturally occurring actin mutants have been reported. This raises some concerns about the wide use of fusion constructs where actin is expressed fused to a fluorescent molecule for imaging purposes (Aizawa et al. 1997; Brault et al. 1999). For instance, observations in fission yeast have revealed detrimental effects on contractile ring formation and cell division by expressing green fluorescent protein fused to the actin N-terminus (Wu and Pollard 2005), and short peptide insertions at different positions in the molecule aimed for so-called FlAsH-labelling (Adams et al. 2002; Martin et al. 2005) all interfered with the proper distribution of the protein (Chen et al. 2012). This is likely to reflect constraints on the molecule that may affect both domain motions and interactions with specific actin binding proteins. Furthermore, expression of modified actins may also interfere with folding and protein quality control mechanisms operated by the molecular chaperone CCT (see below). In addition to fusion constructs to visualize actin distribution in living cells, the fine-tuned operation of the actin motor is often manipulated by drug treatments targeted to intervene with filament turnover in various ways. Although proven to be useful in many situations, some concern has again to be raised since these compounds may also influence actin activities other than filament dynamics. The commonly used latrunculins (A/B) for instance may influence the binding of the modified actin with profilin, ADF/cofilin and thymosin and thereby alter cellular actin organization (Bernstein et al. 2006; Pendleton et al. 2003) see also discussion by (Kudryashov et al. 2010).

The text above is primarily focused on actin control at the periphery of fibroblast-like cells which typically form a broad lamellipod with associated filopodia at their leading edge upon migration. However, the actin motor also operates in cells that attain a less flattened, sometimes more or less rounded morphology with intensive membrane blebbing and a migratory pattern that often is referred to as amoeboid-like (Charras and Paluch 2008; Fackler and Grosse 2008). Lymphatic cells, penetrating tissue borders such as across blood vessel walls or into lymph nodes represent one variant on this theme (Lammermann and Sixt 2009), which is typical also for many invasive cancer cells. In such cases actomyosin-driven contractions at the rear appear to play a more prominent role than during fibroblast-like cell movement, and cell polarization is not always obvious (Lorentzen et al. 2011). Yet polymerization-dependent remodelling of the microfilament arrangement is also important in this context and is influenced by a variety of actin-binding proteins. Also microfilament arrangements classically recognized as “stable” such as in the cell-cell contacts of epithelial adherens junctions or in nerve synapses and therefore not considered to be dynamic are now realized to be formed by highly dynamic filament assemblies (Matus 2000; Yamada and Nelson 2007) the control of which in addition involves subsets of the proteins mentioned above.

### Folding and protein quality control

In addition to the array of proteins that control the dynamic state of the microfilament system, actin also interacts with several molecular chaperones during its biogenesis. The general folding requirements of many newly translated polypeptides are met by the non-specific molecular chaperone Hsc70, which is thought to act either in a passive manner shielding aggregation-prone regions of sequence until folding to the native state can proceed or by inducing local unfolding of misfolded proteins (Mayer and Bukau 2005). However the folding requirements of actin are more stringent, involving interactions with two molecular chaperones that have a restricted range of substrates: prefoldin and chaperonin containing TCP-1 (CCT). Interestingly, tubulin, whilst sharing no structural similarities with actin, also interacts with prefoldin and requires interactions with CCT in order to attain its native conformation (Lundin et al. 2010). Therefore the folding requirements of actin and tubulin result in an interdependency between CCT and the two major force-generating systems in the cell, making CCT essential for virtually all cell motility processes. Here the focus will be on CCT:actin interactions only; for a more comprehensive view of the range of CCT-binding proteins and the debate regarding substrate specificity, see (Dekker et al. 2008; Grantham 2010; Yam et al. 2008).

It has been observed in *S. cerevisiae*, that most of the newly synthesized actin is not able to bind to a chaperone trap, a molecular chaperone that is able to bind to, but not release non-native proteins, (Siegers et al. 1999). This indicates that the unfolded actin is sequestered by chaperones rather than being free in bulk solution and is consistent with the handover of actin occurring between chaperone systems which would result in little actin being vulnerable to aggregation.

Prefoldin (GimC in *S. cerevisiae*) is an oligomer formed from six subunit species in eukaryotes that delivers actin to CCT (Vainberg et al. 1998). Prefoldin binds to actin co-translationally (Hansen et al. 1999) and then most likely assists with the docking of full length actin onto the CCT oligomer: images of prefoldin bound to CCT indicate that binding occurs in an orientation consistent with that observed for actin bound to CCT (Llorca et al. 1999; Martin-Benito et al. 2002).

**Fig. 3:**
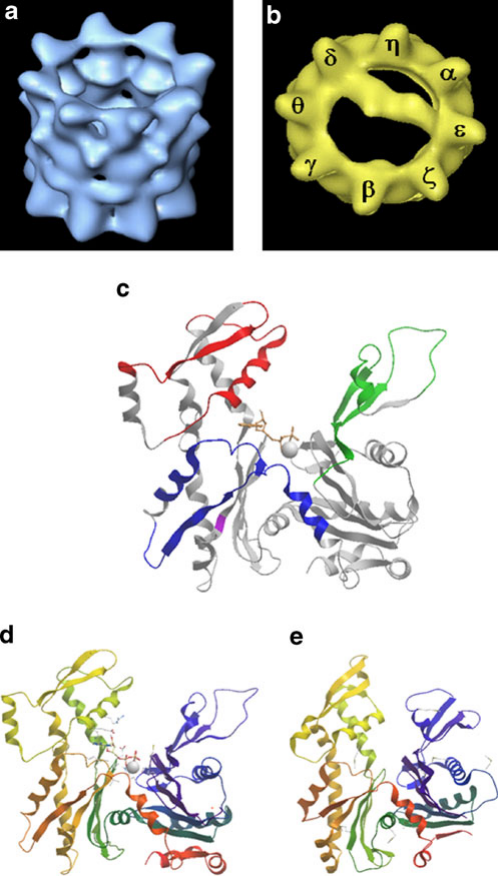
Actin:CCT interactions. Panel **a** shows a 3 dimensional reconstruction of the empty CCT oligomer (Llorca et al. 2001) and in **b** with actin bound (Llorca et al. 1999). Subunits in **b** are labeled as in (Liou and Willison 1997). In **c**, the CCT binding sites in actin as determined by (Hynes and Willison 2000), green, red and blue colors, are shown modeled on to the open structure of actin in profilin:actin (Chik et al. 1996; pdb: 1HLU). The red site contacts either CCTβ or ε and the green CCTδ (see text for details). Position 297 which confers species specificity of yeast CCT:α-actin interactions is shown in purple (Altschuler et al. 2009). In **d** and **e**, the structures of β-actin (**d**) and MreB (**e**) are displayed for comparison (van den Ent et al. 2001; pdb: 1JCE)

The prefoldin genes are not essential in yeast but upon their deletion a reduction in the production of newly folded actin is observed (Siegers et al. 1999). In mice, the loss of prefoldin 1 affects lymphocyte development and also results in conditions associated with ciliary dyskinesia, suggesting that the microfilament and microtubule systems are impaired (Cao et al. 2008). Unlike prefoldin, CCT genes are essential in *S. cerevisiae* (Stoldt et al. 1996) and reductions in CCT subunit levels by siRNA in mammalian cells result in a disordered microfilament system and a halt in cell cycle progression (Grantham et al. 2006). The CCT oligomer (Fig. 3a, b) consists of eight subunits (α to θ in mammalian cells, and 1 to 8 in yeast), which each occupy a fixed position within the chaperonin ring (Dekker et al. 2011; Liou and Willison 1997). Using cryo-electron microscopy and 3D reconstructions, insights have been gained into the mechanism of CCT action during actin folding. Actin binds to CCT in an open conformation via specific CCT subunits (Llorca et al. 1999) and proceeds to a more compact conformation whilst bound to CCT (Llorca et al. 2001). This is consistent with a specific folding intermediate of actin binding to CCT in order to overcome an unfavorable energy barrier, which upon release proceeds to the native state (Altschuler and Willison 2008).

The ability of actin to bind to CCT via two binding sites (as shown using cryo-electron microscopy: Llorca et al. 2000; Llorca et al. 1999) allows CCT to have a mechanical input upon actin during its folding cycle (Fig. 3b) see also discussion by (Grantham 2010). It appears that actin initially binds to CCT via residues located in SD4 (Hynes and Willison 2000; Neirynck et al. 2006), and that the initial capture sites for this interaction are found in the apical substrate binding domains of CCTβ and CCTε (Llorca et al. 1999). After the initial contact, actin is thought to bind to CCTδ via residues located in SD2, resulting in actin being held in an open conformation across the CCT central cavity, (Fig. 3b, c) see also (Hynes and Willison 2000; Llorca et al. 1999; Neirynck et al. 2006).

All CCT subunits contain an ATP-binding site in their equatorial domains and nucleotide-induced conformational changes drive the folding activities of CCT. The binding of ATP to CCT subunits occurs in sequential manner around the chaperonin ring (Lin and Sherman 1997; Rivenzon-Segal et al. 2005). Cryo-electron microscopy of actin:CCT complexes in the presence of AMP-PNP show that the actin is released from the CCTδ subunit under these conditions and forms a more compact structure at one side of the chaperonin cavity (Llorca et al. 2001).

Actin is dependent upon CCT to reach its native state and is insoluble when expressed in *E.coli*. However, when produced at low levels in an *E.coli* lysate in vitro expression system actin forms a soluble folding intermediate which can be processed by CCT to produce native actin (Pappenberger et al. 2006; Stemp et al. 2005). This is an intriguing observation because it highlights both the dependency of actin upon CCT and also demonstrates that the bacterial chaperonin GroEL cannot process the soluble folding intermediate species of actin. It is therefore interesting to note that bacterial and archaeal homologues of actin are able to fold in vivo in the absence of CCT. The actin-like proteins in prokaryotes show high structural similarity to eukaryotic actin (Fig. 3d, e) and see (Roeben et al. 2006; van den Ent et al. 2001). A comparison of the CCT binding sites identified within actin (Hynes and Willison 2000) with the corresponding sequences of MreB reveals that the CCT binding sites on actin are disrupted in MreB (Table 1). Considering this structural similarity and the ability of the actin-like proteins to fold in bacteria, where CCT is absent, it is intriguing that actin cannot reach its native state in the absence of CCT (Pappenberger et al. 2006; Stemp et al. 2005).

**Table 1:** MreB is different in regions corresponding to CCT-interacting sequences in actin

Actin	MreB
43–49	Deleted
60–61	Insertion
64–65	Insertion
196–197	Insertion
197–204	Deleted
231–234	Deleted
243–244	Insertion
321–326	Deleted

Numbers denote stretches of the actin sequence that are located within CCT-binding sites (Hynes and Willison 2000), which are perturbed in MreB

A plausible explanation is that during evolution actin:CCT interactions whilst not essential for the folding of actin, were beneficial either for the speed of folding or for producing a pool of high quality actin monomers that were functionally favorable to the cell. It is therefore possible to imagine that if actin folding was more efficient when assisted by CCT, then interaction sites may have evolved to promote such binding at the expense of actin losing its ability to fold without CCT. This is consistent with the suggestion that CCT has co-evolved with its major substrates actin and tubulin (Archibald et al. 2000).

The dependency of actin upon CCT is illustrated by the species incompatibility between CCT and actin. Whilst the non-muscle β-actin is able to fold to its native state in *S. cerevisiae* (Karlsson 1988; Schűler et al. 2006) skeletal α-actin cannot (Rubenstein 1990). This is explained by the discovery that yeast CCT is unable to fold α-actin and the residue N297 in α-actin is responsible for this folding incompatibility, on the other hand mammalian CCT is able to fold both yeast and α-actin (Altschuler et al. 2009). This demonstrates a difference between yeast and mammalian CCT, which, if explained at molecular level, may shed light on the folding mechanism and how the CCT system has evolved.

Although residue N297 is not located directly within a previously identified CCT binding site (Hynes and Willison 2000; Neirynck et al. 2006), it supports the view that actin:CCT interactions are specific, rather than occurring via more general hydrophobic interactions. Although some substrates of CCT may bind to CCT via hydrophobic sites (Spiess et al. 2006) there is a substantial amount of evidence that CCT:actin interactions are specific (Hynes and Willison 2000; Llorca et al. 1999; Pappenberger et al. 2002).

In addition to the folding requirements of newly-synthesized actin monomers, it is now becoming clear that CCT also has a role extending to the organization of the microfilament system. The CCT oligomer reduces the elongation rate during polymerization in vitro while the final steady state level of filament formation remained unchanged, demonstrating an interaction with already folded actin (Grantham et al. 2002). The fact that the actin filament severing and capping protein gelsolin associates with CCT, emphasizes that CCT may play a more complex role in the control of microfilament dynamics than previously thought (Brackley and Grantham 2011). Not surprisingly, reduction in CCT levels by siRNA resulted in a severely disturbed actin organization, a reduction in the amount of folded monomeric actin as monitored by DNase I-binding, and altered cell morphology and motility (Grantham et al. 2006). Consistently, the mutation G345D in budding yeast CCT4 (CCTδ) results in an abnormal actin cytoskeleton (Vinh and Drubin 1994), apparently due to a slower folding of the actin (Shimon et al. 2008). In yeast, damaged proteins are retained in the mother cell during budding (Aguilaniu et al. 2003). This requires an intact microfilament system (Erjavec et al. 2007), and CCT oligomers purified from a mutant yeast (*sir2Δ*), which is less efficient in this damage segregation, produces native actin more slowly than CCT purified from wild-type cells (Liu et al. 2010). Thus, it would appear that the production rate of newly folded actin impacts on the integrity of the microfilament system.

Another role for CCT with respect to microfilament function involves unassembled CCT subunits. In mammalian cells, altered levels of monomeric CCT subunits influence cell shape, and it has been suggested that the CCTε subunit, which also was shown to associate with stress fibres, is central to this (Brackley and Grantham 2010). Interestingly in a study of yeast CCT subunits, equivalent mutations in the highly-conserved ATP binding sites of each of the subunits resulted in remarkably different phenotypes, such as increase in cell size and sensitivity to latrunculin A (Amit et al. 2010). Although this could reflect an effect on actin folding it is also consistent with the concept that individual CCT subunits have specific functions outside their role in the oligomer.

It is possible that monomeric CCT subunits have chaperone-like activity as has been reported for CCT6 (Kabir et al. 2005) however the binding of individual subunits to actin structures may also reflect an interplay between the folding machinery and the microfilament system in order to coordinate oligomer levels with actin turnover. Clearly future investigations are required to resolve this.

Here we have highlighted the stringent folding requirements for newly synthesized actin and key features of this dynamic force-generating motor juxtaposed to the plasma membrane at advancing cell edges. The intricate control of its organization, its importance for communication between cells and for directed cell movements are essential for all eukaryotes during homeostasis. A full understanding of these processes will therefore be critical for addressing numerous severe pathological conditions such as immunological and neurological diseases as well as cancer.

The literature on actin organization and control is vast. In many cases we have chosen to cite review articles instead of original papers to aid newcomers to the field. We apologize to those authors who have not been cited.

## References

[CR1] Adams SR, Campbell RE, Gross LA, Martin BR, Walkup GK, Yao Y, Llopis J, Tsien RY (2002). New biarsenical ligands and tetracysteine motifs for protein labeling in vitro and in vivo: synthesis and biological applications. J Am Chem Soc.

[CR2] Aguilaniu H, Gustafsson L, Rigoulet M, Nyström T (2003). Asymmetric inheritance of oxidativly damaged proteins during cytokinesis. Science.

[CR3] Aizawa H, Sameshima M, Yahara I (1997). A green fluorescent protein-actin fusion protein dominantly inhibits cytokinesis, cell spreading, and locomotion in Dictyostelium. Cell Struct Funct.

[CR4] Almagro S, Durmort C, Chervin-Petinot A, Heyraud S, Dubois M, Lambert O, Maillefaud C, Hewat E, Schaal JP, Huber P, Gulino-Debrac D (2010). The motor protein myosin-X transports VE-cadherin along filopodia to allow the formation of early endothelial cell-cell contacts. Mol Cell Biol.

[CR5] Altschuler GM, Willison KR (2008). Development of free-energy-based models for chaperonin containing TCP-1 mediated folding of actin. J R Soc Interface.

[CR6] Altschuler GM, Dekker C, McCormack EA, Morris EP, Klug DR, Willison KR (2009). A single amino acid residue is responsible for species-specific incompatibility between CCT and a-actin. FEBS Lett.

[CR7] Amit M, Weisberg SJ, Nadler-Holly M, McCormack EA, Feldmesser E, Kaganovich D, Willison KR, Horovitz A (2010). Equivalent mutations in the eight subunits of the chaperonin CCT produce dramatically different cellular and gene expression phenotypes. J Mol Biol.

[CR8] Archibald JM, Logsdon JM, Doolittle WF (2000). Origin and evolution of eukaryotic chaperonins: phylogenetic evidence for ancient duplications in CCT genes. Mol Biol Evol.

[CR9] Arjonen A, Kaukonen R, Ivaska J (2011). Filopodia and adhesion in cancer cell motility. Cell Adh Migr.

[CR10] Bae YH, Ding Z, Das T, Wells A, Gertler F, Roy P (2010). Profilin1 regulates PI(3,4)P2 and lamellipodin accumulation at the leading edge thus influencing motility of MDA-MB-231 cells. Proc Natl Acad Sci USA.

[CR11] Bamburg JR (1999). Proteins of the ADF/cofilin family: essential regulators of actin dynamics. Annu Rev Cell Dev Biol.

[CR12] Bennett V, Healy J (2008). Organizing the fluid membrane bilayer: diseases linked to spectrin and ankyrin. Trends Mol Med.

[CR13] Bernstein BW, Bamburg JR (2003). Actin-ATP hydrolysis is a major energy drain for neurons. J Neurosci.

[CR14] Bernstein BW, Chen H, Boyle JA, Bamburg JR (2006). Formation of actin-ADF/cofilin rods transiently retards decline of mitochondrial potential and ATP in stressed neurons. Am J Physiol Cell Physiol.

[CR15] Blanchoin L, Pollard TD, Hitchcock-DeGregori SE (2001). Inhibition of the Arp2/3 complex-nucleated actin polymerization and branch formation by tropomyosin. Curr Biol.

[CR16] Blikstad I, Carlsson L (1982). On the dynamics of the microfilament system in HeLa cells. J Cell Biol.

[CR17] Boquet I, Boujemaa R, Carlier MF, Preat T (2000). Ciboulot regulates actin assembly during Drosophila brain metamorphosis. Cell.

[CR18] Bork P, Sander C, Valencia A (1992). An ATPase domain common to prokaryotic cell cycle proteins, sugar kinases, actin, and hsp70 heat shock proteins. Proc Natl Acad Sci USA.

[CR19] Brackley KI, Grantham J (2010). Subunits of the chaperonin CCT interact with F-actin and influence cell shape and cytoskeletal assembly. Exp Cell Res.

[CR20] Brackley KI, Grantham J (2011). Interactions between the actin filament capping and severing protein gelsolin and the molecular chaperone CCT: evidence for nonclassical substrate interactions. Cell Stress Chaperones.

[CR21] Brault V, Sauder U, Reedy MC, Aebi U, Schoenenberger CA (1999). Differential epitope tagging of actin in transformed Drosophila produces distinct effects on myofibril assembly and function of the indirect flight muscle. Mol Biol Cell.

[CR22] Breitsprecher D, Kiesewetter AK, Linkner J, Vinzenz M, Stradal TE, Small JV, Curth U, Dickinson RB, Faix J (2011). Molecular mechanism of Ena/VASP-mediated actin-filament elongation. EMBO J.

[CR23] Bugyi B, Carlier MF (2010). Control of actin filament treadmilling in cell motility. Annu Rev Biophys.

[CR24] Bugyi B, Didry D, Carlier MF (2010). How tropomyosin regulates lamellipodial actin-based motility: a combined biochemical and reconstituted motility approach. EMBO J.

[CR25] Campellone KG, Welch MD (2010). A nucleator arms race: cellular control of actin assembly. Nat Rev Mol Cell Biol.

[CR26] Cao S, Carlesso G, Osipovich AB, Llanes J, Lin Q, Hoek KL, Khan WN, Ruley HE (2008). Subunit 1 of the prefoldin chaperone complex is required for lymphocyte development and function. J Immunol.

[CR27] Carlier MF, Jean C, Rieger KJ, Lenfant M, Pantaloni D (1993). Modulation of the interaction between G-actin and thymosin beta 4 by the ATP/ADP ratio: possible implication in the regulation of actin dynamics. Proc Natl Acad Sci USA.

[CR28] Carlier MF, Husson C, Renault L, Didry D (2011). Control of actin assembly by the WH2 domains and their multifunctional tandem repeats in Spire and Cordon-Bleu. Int Rev Cell Mol Biol.

[CR29] Castellano F, Le Clainche C, Patin D, Carlier MF, Chavrier P (2001). A WASp-VASP complex regulates actin polymerization at the plasma membrane. EMBO J.

[CR30] Chan KT, Creed SJ, Bear JE (2011). Unraveling the enigma: progress towards understanding the coronin family of actin regulators. Trends Cell Biol.

[CR31] Chang F, Drubin D, Nurse P (1997). cdc12p, a protein required for cytokinesis in fission yeast, is a component of the cell division ring and interacts with profilin. J Cell Biol.

[CR32] Charras G, Paluch E (2008). Blebs lead the way: how to migrate without lamellipodia. Nat Rev Mol Cell Biol.

[CR33] Chaudhry F, Little K, Talarico L, Quintero-Monzon O, Goode BL (2010). A central role for the WH2 domain of Srv2/CAP in recharging actin monomers to drive actin turnover in vitro and in vivo. Cytoskeleton (Hoboken)..

[CR34] Chen Q, Nag S, Pollard TD (2012). Formins filter modified actin subunits during processive elongation. J Struct Biol.

[CR35] Chik JK, Lindberg U, Schutt CE (1996). The structure of an open state of beta-actin at 2.65 A resolution. J Mol Biol.

[CR36] Dekker C, Stirling PC, McCormack EA, Filmore H, Paul A, Brost RL, Costanzo M, Boone C, Leroux MR, Willison KR (2008). The interaction network of the chaperonin CCT. EMBO J.

[CR37] Dekker C, Roe SM, McCormack EA, Beuron F, Pearl LH, Willison KR (2011). The crystal structure of yeast CCT reveals intrinsic asymmetry of eukaryotic cytosolic chaperonins. EMBO J.

[CR38] Depetris RS, Wu J, Hubbard SR (2009). Structural and functional studies of the Ras-associating and pleckstrin-homology domains of Grb10 and Grb14. Nat Struct Mol Biol.

[CR39] Didry D, Cantrelle FX, Husson C, Roblin P, Moorthy AM, Perez J, Le Clainche C, Hertzog M, Guittet E, Carlier MF, van Heijenoort C, Renault L (2011). How a single residue in individual beta-thymosin/WH2 domains controls their functions in actin assembly. EMBO J.

[CR40] Ding Z, Lambrechts A, Parepally M, Roy P (2006). Silencing profilin-1 inhibits endothelial cell proliferation, migration and cord morphogenesis. J Cell Sci.

[CR41] Dominguez R (2007). The beta-thymosin/WH2 fold: multifunctionality and structure. Ann N Y Acad Sci.

[CR42] Dominguez R (2010). Structural insights into de novo actin polymerization. Curr Opin Struct Biol.

[CR43] Dominguez R, Holmes KC (2011). Actin structure and function. Annu Rev Biophys.

[CR44] Erjavec N, Larsson L, Grantham J, Nyström T (2007). Accelerated aging and failure to segregate damaged proteins in Sir2 mutants can be suppressed by overproducing the protein aggregation-remodelling factor Hsp104. Genes Dev.

[CR45] Evangelista M, Pruyne D, Amberg DC, Boone C, Bretscher A (2002). Formins direct Arp2/3-independent actin filament assembly to polarize cell growth in yeast. Nat Cell Biol.

[CR46] Fackler OT, Grosse R (2008). Cell motility through plasma membrane blebbing. J Cell Biol.

[CR47] Flaherty KM, McKay DB, Kabsch W, Holmes KC (1991). Similarity of the three-dimensional structures of actin and the ATPase fragment of a 70-kDa heat shock cognate protein. Proc Natl Acad Sci USA.

[CR48] Fujii T, Iwane AH, Yanagida T, Namba K (2010). Direct visualization of secondary structures of F-actin by electron cryomicroscopy. Nature.

[CR49] Galbraith CG, Yamada KM, Galbraith JA (2007). Polymerizing actin fibers position integrins primed to probe for adhesion sites. Science.

[CR50] Galkin VE, Orlova A, Schroder GF, Egelman EH (2010). Structural polymorphism in F-actin. Nat Struct Mol Biol.

[CR51] Galkin VE, Orlova A, Egelman EH (2012). Actin filaments as tension sensors. Curr Biol.

[CR52] Gandhi M, Smith BA, Bovellan M, Paavilainen V, Daugherty-Clarke K, Gelles J, Lappalainen P, Goode BL (2010). GMF is a cofilin homolog that binds Arp2/3 complex to stimulate filament debranching and inhibit actin nucleation. Curr Biol.

[CR53] Geiger B, Yamada KM (2011). Molecular architecture and function of matrix adhesions. Cold Spring Harb Perspect Biol.

[CR54] Goley ED, Welch MD (2006). The ARP2/3 complex: an actin nucleator comes of age. Nat Rev Mol Cell Biol.

[CR55] Grantham J, Durante P, Colucci L (2010). The eukaryotic chaperonin CCT (TRiC):structure, mechanisms of action and substrate diversity. Molecular chaperones: roles structures and mechanisms.

[CR56] Grantham J, Ruddock LW, Roobol A, Carden MJ (2002). Eukaryotic chaperonin containing T-complex polypeptide 1 interacts with filamentous actin and reduces the initial rate of actin polymerization in vitro. Cell Stress Chaperones.

[CR57] Grantham J, Brackley KI, Willison KR (2006). Substantial CCT activity is required for cell cycle progression and cytoskeletal organization in mammalian cells. Exp Cell Res.

[CR58] Grenklo S, Geese M, Lindberg U, Wehland J, Karlsson R, Sechi AS (2003). A crucial role for profilin-actin in the intracellular motility of Listeria monocytogenes. EMBO Rep.

[CR59] Grenklo S, Hillberg L, Zhao Rathje LS, Pinaev G, Schutt CE, Lindberg U (2008). Tropomyosin assembly intermediates in the control of microfilament system turnover. Eur J Cell Biol.

[CR60] Hajkova L, Nyman T, Lindberg U, Karlsson R (2000). Effects of cross-linked profilin:beta/gamma-actin on the dynamics of the microfilament system in cultured cells. Exp Cell Res.

[CR61] Hansen SD, Mullins RD (2010). VASP is a processive actin polymerase that requires monomeric actin for barbed end association. J Cell Biol.

[CR62] Hansen WJ, Cowan NJ, Welch WJ (1999). Prefoldin-nascent chain complexes in the folding of cytoskeletal proteins. J Cell Biol.

[CR63] Hernandez-Varas P, Colo GP, Bartolome RA, Paterson A, Medrano-Fernandez I, Arellano-Sanchez N, Cabanas C, Sanchez-Mateos P, Lafuente EM, Boussiotis VA, Stromblad S, Teixido J (2011). Rap1-GTP-interacting adaptor molecule (RIAM) protein controls invasion and growth of melanoma cells. J Biol Chem.

[CR64] Hertzog M, Yarmola EG, Didry D, Bubb MR, Carlier MF (2002). Control of actin dynamics by proteins made of beta-thymosin repeats: the actobindin family. J Biol Chem.

[CR65] Hertzog M, van Heijenoort C, Didry D, Gaudier M, Coutant J, Gigant B, Didelot G, Preat T, Knossow M, Guittet E, Carlier MF (2004). The beta-thymosin/WH2 domain; structural basis for the switch from inhibition to promotion of actin assembly. Cell.

[CR66] Hillberg L, Zhao Rathje LS, Nyakern-Meazza M, Helfand B, Goldman RD, Schutt CE, Lindberg U (2006). Tropomyosins are present in lamellipodia of motile cells. Eur J Cell Biol.

[CR67] Hilpela P, Vartiainen MK, Lappalainen P (2004). Regulation of the actin cytoskeleton by PI(4,5)P2 and PI(3,4,5)P3. Curr Top Microbiol Immunol.

[CR68] Hoglund AS, Karlsson R, Arro E, Fredriksson BA, Lindberg U (1980). Visualization of the peripheral weave of microfilaments in glia cells. J Muscle Res Cell Motil.

[CR69] Husson C, Cantrelle FX, Roblin P, Didry D, Le KH, Perez J, Guittet E, Van Heijenoort C, Renault L, Carlier MF (2010). Multifunctionality of the beta-thymosin/WH2 module: G-actin sequestration, actin filament growth, nucleation, and severing. Ann N Y Acad Sci.

[CR70] Hynes GM, Willison KR (2000). Individual subunits of the eukaryotic cytosolic chaperonin mediate interactions with binding sites located on subdomains of β-actin. J Biol Chem.

[CR71] Ito T, Narita A, Hirayama T, Taki M, Iyoshi S, Yamamoto Y, Maeda Y, Oda T (2011). Human spire interacts with the barbed end of the actin filament. J Mol Biol.

[CR72] Janke J, Schluter K, Jandrig B, Theile M, Kolble K, Arnold W, Grinstein E, Schwartz A, Estevez-Schwarz L, Schlag PM, Jockusch BM, Scherneck S (2000). Suppression of tumorigenicity in breast cancer cells by the microfilament protein profilin 1. J Exp Med.

[CR73] Jenzora A, Behrendt B, Small JV, Wehland J, Stradal TE (2006). PREL1 provides a link from Ras signalling to the actin cytoskeleton via Ena/VASP proteins. FEBS Lett.

[CR74] Kabir MA, Kaminska J, Segel GB, Bethlendy G, Lin P, Seta FD, Blegen C, Swiderek KM, Zoladek T, Arndt KT, Sherman F (2005). Physiological effects of unassembled chaperonin Cct subunits in the yeast *Saccharomyces cerevisiae*. Yeast.

[CR75] Kabsch W, Mannherz HG, Suck D, Pai EF, Holmes KC (1990). Atomic structure of the actin:DNase I complex. Nature.

[CR76] Karlsson R (1988). Expression of chicken beta-actin in Saccharomyces cerevisiae. Gene.

[CR77] Karlsson R, Lindberg U, Lappalainen P (2007). Profilin, an essential control element for actin polymerization. Actin Monomer-binding Proteins.

[CR78] Kerber ML, Jacobs DT, Campagnola L, Dunn BD, Yin T, Sousa AD, Quintero OA, Cheney RE (2009). A novel form of motility in filopodia revealed by imaging myosin-X at the single-molecule level. Curr Biol.

[CR79] Keren K, Yam PT, Kinkhabwala A, Mogilner A, Theriot JA (2009). Intracellular fluid flow in rapidly moving cells. Nat Cell Biol.

[CR80] Koestler SA, Auinger S, Vinzenz M, Rottner K, Small JV (2008). Differentially oriented populations of actin filaments generated in lamellipodia collaborate in pushing and pausing at the cell front. Nat Cell Biol.

[CR81] Koestler SA, Rottner K, Lai F, Block J, Vinzenz M, Small JV (2009). F- and G-actin concentrations in lamellipodia of moving cells. PLoS One.

[CR82] Korenbaum E, Nordberg P, Bjorkegren-Sjogren C, Schutt CE, Lindberg U, Karlsson R (1998). The role of profilin in actin polymerization and nucleotide exchange. Biochemistry.

[CR83] Korn ED (1982). Actin polymerization and its regulation by proteins from nonmuscle cells. Physiol Rev.

[CR84] Korn ED, Carlier MF, Pantaloni D (1987). Actin polymerization and ATP hydrolysis. Science.

[CR85] Krause M, Leslie JD, Stewart M, Lafuente EM, Valderrama F, Jagannathan R, Strasser GA, Rubinson DA, Liu H, Way M, Yaffe MB, Boussiotis VA, Gertler FB (2004). Lamellipodin, an Ena/VASP ligand, is implicated in the regulation of lamellipodial dynamics. Dev Cell.

[CR86] Kudryashov DS, Grintsevich EE, Rubenstein PA, Reisler E (2010). A nucleotide state-sensing region on actin. J Biol Chem.

[CR87] Kursula P, Kursula I, Massimi M, Song YH, Downer J, Stanley WA, Witke W, Wilmanns M (2008). High-resolution structural analysis of mammalian profilin 2a complex formation with two physiological ligands: the formin homology 1 domain of mDia1 and the proline-rich domain of VASP. J Mol Biol.

[CR88] Lafuente EM, van Puijenbroek AA, Krause M, Carman CV, Freeman GJ, Berezovskaya A, Constantine E, Springer TA, Gertler FB, Boussiotis VA (2004). RIAM, an Ena/VASP and Profilin ligand, interacts with Rap1-GTP and mediates Rap1-induced adhesion. Dev Cell.

[CR89] Lai FP, Szczodrak M, Block J, Faix J, Breitsprecher D, Mannherz HG, Stradal TE, Dunn GA, Small JV, Rottner K (2008). Arp2/3 complex interactions and actin network turnover in lamellipodia. EMBO J.

[CR90] Lammermann T, Sixt M (2009). Mechanical modes of ‘amoeboid’ cell migration. Curr Opin Cell Biol.

[CR91] Lassing I, Hillberg L, Hoglund AS, Karlsson R, Schutt C, Lindberg U (2010). Tropomyosin is a tetramer under physiological salt conditions. Cytoskeleton (Hoboken).

[CR92] Le Clainche C, Carlier MF (2008). Regulation of actin assembly associated with protrusion and adhesion in cell migration. Physiol Rev.

[CR93] Lee HS, Lim CJ, Puzon-McLaughlin W, Shattil SJ, Ginsberg MH (2009). RIAM activates integrins by linking talin to ras GTPase membrane-targeting sequences. J Biol Chem.

[CR94] Lin P, Sherman F (1997). The unique hetero-oligomeric nature of the subunits in the catalytic cooperativity of the yeast Cct chaperonin complex. Proc Natl Acad Sci USA.

[CR95] Lindberg U, Karlsson R, Lassing I, Schutt CE, Hoglund AS (2008). The microfilament system and malignancy. Semin Cancer Biol.

[CR96] Liou AK, Willison KR (1997). Elucidation of the subunit orientation in CCT (chaperonin containing TCP1) from the subunit composition of CCT micro-complexes. EMBO J.

[CR97] Liu B, Larsson L, Caballero A, Hao X, Öling D, Grantham J, Nyström T (2010). The polarisome is required for segregation and retrograde transport of protein aggregates. Cell.

[CR98] Llorca O, McCormack EA, Hynes G, Grantham J, Cordell J, Carrascosa JL, Willison KR, Fernandez JJ, Valpuesta JM (1999). Eukaryotic type II chaperonin CCT interacts with actin through specific subunits. Nature.

[CR99] Llorca O, Martin-Benito J, Ritco-Vonsovici M, Grantham J, Hynes GM, Willison KR, Carrascosa JL, Valpuesta JM (2000). Eukaryotic chaperonin CCT stabilizes actin and tubulin folding intermediates in open quasi-native conformations. EMBO J.

[CR100] Llorca O, Martin-Benito J, Grantham J, Ritco-Vonsovici M, Willison KR, Carrascosa JL, Valpuesta JM (2001). The ‘sequential allosteric ring’ mechanism in the eukaryotic chaperonin- assisted folding of actin and tubulin. EMBO J.

[CR101] Lorentzen A, Bamber J, Sadok A, Elson-Schwab I, Marshall CJ (2011). An ezrin-rich, rigid uropod-like structure directs movement of amoeboid blebbing cells. J Cell Sci.

[CR102] Lundin VF, Leroux MR, Stirling PC (2010). Quality control of cytoskeletal proteins and human disease. Trends Biochem Sci.

[CR103] Martin BR, Giepmans BN, Adams SR, Tsien RY (2005). Mammalian cell-based optimization of the biarsenical-binding tetracysteine motif for improved fluorescence and affinity. Nat Biotechnol.

[CR104] Martin-Benito J, Boskovic J, Gomez-Puertas P, Carrascosa JL, Simons CT, Lewis SA, Bartolini F, Cowan NJ, Valpuesta JM (2002). Structure of eukaryotic prefoldin and of its complexes with unfolded actin and the cytosolic chaperonin CCT. EMBO J.

[CR105] Matsudaira P (1994). The fimbrin and alpha-actinin footprint on actin. J Cell Biol.

[CR106] Mattila PK, Salminen M, Yamashiro T, Lappalainen P (2003). Mouse MIM, a tissue-specific regulator of cytoskeletal dynamics, interacts with ATP-actin monomers through its C-terminal WH2 domain. J Biol Chem.

[CR107] Matus A (2000). Actin-based plasticity in dendritic spines. Science.

[CR108] Mayer MP, Bukau B (2005). Hsp70 chaperones: cellular functions and molecular mechanism. Cell Mol Life Sci.

[CR109] Michael M, Vehlow A, Navarro C, Krause M (2010). c-Abl, Lamellipodin, and Ena/VASP proteins cooperate in dorsal ruffling of fibroblasts and axonal morphogenesis. Curr Biol.

[CR110] Michelot A, Drubin DG (2011). Building distinct actin filament networks in a common cytoplasm. Curr Biol.

[CR111] Miki H, Suetsugu S, Takenawa T (1998). WAVE, a novel WASP-family protein involved in actin reorganization induced by Rac. EMBO J.

[CR112] Mockrin SC, Korn ED (1980). Acanthamoeba profilin interacts with G-actin to increase the rate of exchange of actin-bound adenosine 5′-triphosphate. Biochemistry.

[CR113] Moriyama K, Yahara I (2002). Human CAP1 is a key factor in the recycling of cofilin and actin for rapid actin turnover. J Cell Sci.

[CR114] Mullins RD, Heuser JA, Pollard TD (1998). The interaction of Arp2/3 complex with actin: nucleation, high affinity pointed end capping, and formation of branching networks of filaments. Proc Natl Acad Sci USA.

[CR115] Nakamura F, Stossel TP, Hartwig JH (2011). The filamins: organizers of cell structure and function. Cell Adh Migr.

[CR116] Neirynck K, Waterschoot D, Vandekerckhove J, Ampe C, Rommelaere H (2006). Actin interacts with CCT via discrete binding sites: a binding transition-release model for CCT-mediated actin folding. J Mol Biol.

[CR117] Neisch AL, Fehon RG (2011). Ezrin, Radixin and Moesin: key regulators of membrane-cortex interactions and signaling. Curr Opin Cell Biol.

[CR118] Nyman T, Page R, Schutt CE, Karlsson R, Lindberg U (2002). A cross-linked profilin-actin heterodimer interferes with elongation at the fast-growing end of F-actin. J Biol Chem.

[CR119] O’Neill GM, Stehn J, Gunning PW (2008). Tropomyosins as interpreters of the signalling environment to regulate the local cytoskeleton. Semin Cancer Biol.

[CR120] Oda T, Maeda Y (2010). Multiple Conformations of F-actin. Structure.

[CR121] Okabe S, Hirokawa N (1989). Incorporation and turnover of biotin-labeled actin microinjected into fibroblastic cells: an immunoelectron microscopic study. J Cell Biol.

[CR122] Ono S, Ono K (2002). Tropomyosin inhibits ADF/cofilin-dependent actin filament dynamics. J Cell Biol.

[CR123] Otey CA, Carpen O (2004). Alpha-actinin revisited: a fresh look at an old player. Cell Motil Cytoskeleton.

[CR124] Pantaloni D, Carlier MF (1993). How profilin promotes actin filament assembly in the presence of thymosin beta 4. Cell.

[CR125] Pappenberger G, Wilsher JA, Roe SM, Counsell DJ, Willison KR, Pearl LH (2002). Crystal structure of the CCTgamma apical domain: implications for substrate binding to the eukaryotic cytosolic chaperonin. J Mol Biol.

[CR126] Pappenberger G, McCormack EA, Willison KR (2006). Quantitative actin folding reactions using yeast CCT purified via an internal tag in the CCT3/gamma subunit. J Mol Biol.

[CR127] Patsoukis N, Lafuente EM, Meraner P, Kim J, Dombkowski D, Li L, Boussiotis VA (2009). RIAM regulates the cytoskeletal distribution and activation of PLC-gamma1 in T cells. Sci Signal.

[CR128] Paul AS, Pollard TD (2009). Review of the mechanism of processive actin filament elongation by formins. Cell Motil Cytoskeleton.

[CR129] Pendleton A, Pope B, Weeds A, Koffer A (2003). Latrunculin B or ATP depletion induces cofilin-dependent translocation of actin into nuclei of mast cells. J Biol Chem.

[CR130] Pollard TD, Borisy GG (2003). Cellular motility driven by assembly and disassembly of actin filaments. Cell.

[CR131] Pollard TD, Blanchoin L, Mullins RD (2000). Molecular mechanisms controlling actin filament dynamics in nonmuscle cells. Annu Rev Biophys Biomol Struct.

[CR132] Porta JC, Borgstahl GE (2012). Structural basis for profilin-mediated actin nucleotide exchange. J Mol Biol.

[CR133] Poukkula M, Kremneva E, Serlachius M, Lappalainen P (2011). Actin-depolymerizing factor homology domain: a conserved fold performing diverse roles in cytoskeletal dynamics. Cytoskeleton (Hoboken).

[CR134] Prochniewicz E, Zhang Q, Janmey PA, Thomas DD (1996). Cooperativity in F-actin: binding of gelsolin at the barbed end affects structure and dynamics of the whole filament. J Mol Biol.

[CR135] Reinhard M, Giehl K, Abel K, Haffner C, Jarchau T, Hoppe V, Jockusch BM, Walter U (1995). The proline-rich focal adhesion and microfilament protein VASP is a ligand for profilins. EMBO J.

[CR136] Renault L, Bugyi B, Carlier MF (2008). Spire and Cordon-bleu: multifunctional regulators of actin dynamics. Trends Cell Biol.

[CR137] Rivenzon-Segal D, Wolf SG, Shimon L, Willison KR, Horovitz A (2005). Sequential ATP-induced allosteric transitions of the cytoplasmic chaperonin containing TCP-1 revealed by EM analysis. Nat Struct Mol Biol.

[CR138] Rivera CR, Kollman JM, Polka JK, Agard DA, Mullins RD (2011). Architecture and assembly of a divergent member of the ParM family of bacterial actin-like proteins. J Biol Chem.

[CR139] Robinson RC, Turbedsky K, Kaiser DA, Marchand JB, Higgs HN, Choe S, Pollard TD (2001). Crystal structure of Arp2/3 complex. Science.

[CR140] Roeben A, Kofler C, Nagy I, Nickell S, Hartl FU, Bracher A (2006). Crystal structure of an archaeal actin homolog. J Mol Biol.

[CR141] Rottner K, Hanisch J, Campellone KG (2010). WASH, WHAMM and JMY: regulation of Arp2/3 complex and beyond. Trends Cell Biol.

[CR142] Rubenstein PA (1990). The functional importance of multiple actin isoforms. Bioessays.

[CR143] Safer D, Elzinga M, Nachmias VT (1991). Thymosin beta 4 and Fx, an actin-sequestering peptide, are indistinguishable. J Biol Chem.

[CR144] Schevzov G, Whittaker SP, Fath T, Lin JJ, Gunning PW (2011). Tropomyosin isoforms and reagents. Bioarchitecture..

[CR145] Schűler H (2001). ATPase activity and conformational changes in the regulation of actin. Biochim Biophys Acta.

[CR146] Schűler H, Karlsson R, Lindberg U, Celis IJ (2006). Purification of non-muscle actin. Cell Biology. A Laboratory Handbook.

[CR147] Schutt CE, Myslik JC, Rozycki MD, Goonesekere NC, Lindberg U (1993). The structure of crystalline profilin-beta-actin. Nature.

[CR148] Shimon L, Hynes GM, McCormack EA, Willison KR, Horovitz A (2008). ATP-induced allostery in the eukaryotic chaperonin CCTis abolished by the mutation G345D in CCT4 that renders yeast temperature sensitive for growth. J Mol Biol.

[CR149] Siegers K, Waldmann T, Leroux MR, Grein K, Shevchenko A, Schiebel E, Hartl FU (1999). Compartmentation of protein folding in vivo: sequestration of non-native polypeptide by the chaperonin-GimC system. EMBO J.

[CR150] Sjoblom B, Salmazo A, Djinovic-Carugo K (2008). Alpha-actinin structure and regulation. Cell Mol Life Sci.

[CR151] Skau CT, Neidt EM, Kovar DR (2009). Role of tropomyosin in formin-mediated contractile ring assembly in fission yeast. Mol Biol Cell.

[CR152] Small JV (1981). Organization of actin in the leading edge of cultured cells: influence of osmium tetroxide and dehydration on the ultrastructure of actin meshworks. J Cell Biol.

[CR153] Small JV (2010). Dicing with dogma: de-branching the lamellipodium. Trends Cell Biol.

[CR154] Small JV, Winkler C, Vinzenz M, Schmeiser C (2011). Reply: Visualizing branched actin filaments in lamellipodia by electron tomography. Nat Cell Biol.

[CR155] Spiess C, Miller EJ, McClellan AJ, Frydman J (2006). Identification of the TRiC/CCT substrate binding sites uncovers the function of subunit diversity in eukaryotic chaperonins. Mol Cell.

[CR156] Splettstoesser T, Holmes KC, Noe F, Smith JC (2011). Structural modeling and molecular dynamics simulation of the actin filament. Proteins.

[CR157] Stehn JR, Schevzov G, O’Neill GM, Gunning PW (2006). Specialisation of the tropomyosin composition of actin filaments provides new potential targets for chemotherapy. Curr Cancer Drug Targets.

[CR158] Stemp MJ, Guha S, Hartl FU, Barral JM (2005). Efficient production of native actin upon translation in a bacterial lysate supplemented with the eukaryotic chaperonin TRiC. Biol Chem.

[CR159] Stoldt V, Rademacher F, Kehren V, Ernst JF, Pearce DA, Sherman F (1996). Review: The Cct eukaryotic chaperonin subunits of Saccharomyces cerevisiae and other yeasts. Yeast.

[CR160] Suetsugu S, Miki H, Takenawa T (1998). The essential role of profilin in the assembly of actin for microspike formation. EMBO J.

[CR161] Svitkina TM, Borisy GG (1999). Arp2/3 complex and actin depolymerizing factor/cofilin in dendritic organization and treadmilling of actin filament array in lamellipodia. J Cell Biol.

[CR162] Takenawa T, Suetsugu S (2007). The WASP-WAVE protein network: connecting the membrane to the cytoskeleton. Nat Rev Mol Cell Biol.

[CR163] Tilney LG, Bonder EM, Coluccio LM, Mooseker MS (1983). Actin from Thyone sperm assembles on only one end of an actin filament: a behavior regulated by profilin. J Cell Biol.

[CR164] Tojkander S, Gateva G, Schevzov G, Hotulainen P, Naumanen P, Martin C, Gunning PW, Lappalainen P (2011). A molecular pathway for myosin II recruitment to stress fibers. Curr Biol.

[CR165] Tokuo H, Ikebe M (2004). Myosin X transports Mena/VASP to the tip of filopodia. Biochem Biophys Res Commun.

[CR166] Urban E, Jacob S, Nemethova M, Resch GP, Small JV (2010). Electron tomography reveals unbranched networks of actin filaments in lamellipodia. Nat Cell Biol.

[CR167] Uyeda TQ, Iwadate Y, Umeki N, Nagasaki A, Yumura S (2011). Stretching actin filaments within cells enhances their affinity for the myosin II motor domain. PLoS One.

[CR168] Vainberg IE, Lewis SA, Rommelaere H, Ampe C, Vandekerckhove J, Klein HL, Cowan NJ (1998). Prefoldin, a chaperone that delivers unfolded proteins to cytosolic chaperonin. Cell.

[CR169] van den Ent F, Amos LA, Lowe J (2001). Prokaryotic origin of the actin cytoskeleton. Nature.

[CR170] van Rheenen J, Song X, van Roosmalen W, Cammer M, Chen X, Desmarais V, Yip SC, Backer JM, Eddy RJ, Condeelis JS (2007). EGF-induced PIP2 hydrolysis releases and activates cofilin locally in carcinoma cells. J Cell Biol.

[CR171] Vinh DB, Drubin DG (1994). A yeast TCP-1-like protein is required for actin function in vivo. Proc Natl Acad Sci.

[CR172] Wang YL (1985). Exchange of actin subunits at the leading edge of living fibroblasts: possible role of treadmilling. J Cell Biol.

[CR173] Watanabe N, Madaule P, Reid T, Ishizaki T, Watanabe G, Kakizuka A, Saito Y, Nakao K, Jockusch BM, Narumiya S (1997). p140mDia, a mammalian homolog of Drosophila diaphanous, is a target protein for Rho small GTPase and is a ligand for profilin. EMBO J.

[CR174] Wawro B, Greenfield NJ, Wear MA, Cooper JA, Higgs HN, Hitchcock-DeGregori SE (2007). Tropomyosin regulates elongation by formin at the fast-growing end of the actin filament. Biochemistry.

[CR175] Wegner A (1976). Head to tail polymerization of actin. J Mol Biol.

[CR176] Wu JQ, Pollard TD (2005). Counting cytokinesis proteins globally and locally in fission yeast. Science.

[CR177] Wu C, Asokan SB, Berginski ME, Haynes EM, Sharpless NE, Griffith JD, Gomez SM, Bear JE (2012). Arp2/3 is critical for lamellipodia and response to extracellular matrix cues but is dispensable for chemotaxis. Cell.

[CR178] Yam AY, Xia Y, Lin H-TJ, Burlingame A, Gerstein M, Frydman J (2008). Defining the TRiC/CCT interactome links chaperonin function to stabilization of newly-made proteins with complex topologies. Nat Struct Mol Biol.

[CR179] Yamada S, Nelson WJ (2007). Synapses: sites of cell recognition, adhesion, and functional specification. Annu Rev Biochem.

[CR180] Yang C, Svitkina T (2011). Visualizing branched actin filaments in lamellipodia by electron tomography. Nat Cell Biol.

[CR181] Yarar D, D’Alessio JA, Jeng RL, Welch MD (2002). Motility determinants in WASP family proteins. Mol Biol Cell.

[CR182] Ydenberg CA, Smith BA, Breitsprecher D, Gelles J, Goode BL (2011). Cease-fire at the leading edge: new perspectives on actin filament branching, debranching, and cross-linking. Cytoskeleton (Hoboken).

[CR183] Zhang H, Berg JS, Li Z, Wang Y, Lang P, Sousa AD, Bhaskar A, Cheney RE, Stromblad S (2004). Myosin-X provides a motor-based link between integrins and the cytoskeleton. Nat Cell Biol.

[CR184] Zhang L, Mao YS, Janmey PA, Yin HL (2012). Phosphatidylinositol 4, 5 bisphosphate and the actin cytoskeleton. Subcell Biochem.

[CR185] Zicha D, Dobbie IM, Holt MR, Monypenny J, Soong DY, Gray C, Dunn GA (2003). Rapid actin transport during cell protrusion. Science.

[CR186] Zou L, Jaramillo M, Whaley D, Wells A, Panchapakesa V, Das T, Roy P (2007). Profilin-1 is a negative regulator of mammary carcinoma aggressiveness. Br J Cancer.

